# Therapeutic Potential of Fucoidan in Alleviating Histamine-Induced Liver Injury: Insights from Mice Studies

**DOI:** 10.3390/foods13101523

**Published:** 2024-05-14

**Authors:** Mengyao Zhang, Huiqian Liu, Linlin Xu, Xizi Zhang, Wei Chen, Chengtao Wang

**Affiliations:** Key Laboratory of Geriatric Nutrition and Health, Ministry of Education, Beijing Advanced Innovation Center for Food Nutrition and Human Health, Beijing Engineering and Technology Research Center of Food Additives, School of Food and Health, Beijing Technology and Business University, Beijing 100048, China; 2230201051@st.btbu.edu.cn (M.Z.); q2223599235@163.com (H.L.); xll18515351711@163.com (L.X.); zhangxizi1999@163.com (X.Z.); weichen@btbu.edu.cn (W.C.)

**Keywords:** fucoidan, histamine, liver injury, gut microbiota

## Abstract

Histamine, a bioactive component in certain foods such as Huangjiu has been associated with liver injury and disrupted intestinal balance. This study explored the potential therapeutic effects of fucoidan (FCD) in mitigating histamine-induced imbalances in mice. We found that FCD mitigated liver injury, reducing transaminases, oxidative stress, and inflammation. Histological improvements included decreased cell infiltration and necrosis. FCD restored tight junction proteins and suppressed inflammation-related genes. Western blot analysis revealed FCD’s impact on TGF-*β*1, *p*-AKT, AKT, CYP2E1, Grp78, NLRP3, Cas-1, and GSDMD. Gut LPS levels decreased with FCD. Gut microbiota analysis showed FCD’s modulation effect, reducing Firmicutes and increasing Bacteroides. FCD demonstrates potential in alleviating histamine-induced liver injury, regulating inflammation, and influencing gut microbiota. Further research exploring higher dosages and additional parameters is warranted.

## 1. Introduction

Biogenic amines (BAs) are substances frequently found in fermented foods derived from protein-rich sources such as meat, eggs, milk, and cereals [1]. While small amounts of BAs are necessary for synthesizing bioactive substances, high concentrations pose health risks [2,3,4]. Key BAs include histamine, cadaverine, tyramine, 2-phenylethylamine, and putrescine [5,6], with histamine being particularly linked to liver and intestinal disorders when it accumulates excessively in the body [7,8,9]. Studies utilizing a *Cyprinus carpio* model suggest that histamine can accelerate poisoning onset in carp [10]. Histamine is known for its slow metabolic rate and its interference with the gastrointestinal processing of other nutrients [11]. In general, histamine concentrations above 35 mg/L trigger adverse reactions in mice [9], initiating an abnormal immune response and the production of excessive free radicals (ROS) in the body. These ROS activate nuclear transcription factors, leading to the activation of Kupffer cells, which in turn enhance cytokine secretion such as TNF-*α* and NO. High levels of TNF-*α* prompt immune cells to release inflammatory cytokines like interleukin 1 (IL-1), interleukin 6 (IL-6), and interleukin 10 (IL-10), exacerbating liver damage and intestinal dysfunction, ultimately leading to oxidative stress-related illnesses [12]. Moreover, recent research has highlighted a significant correlation between liver disorders and intestinal health, noting that elements from the intestinal environment such as bacteria and their metabolic by-products can cross the intestinal barrier, enter the bloodstream, and influence liver metabolism [13,14]. Luo et al. (2022) revealed that oral histamine induced changes in the structure and composition of the intestinal flora, particularly the *Lachnospiraceae_NK4A136_group* and *norank_f_norank_o_clostridia_ug-014*, increasing inflammatory LPS [15], thereby inducing oxidative and inflammatory damage in the liver.

Brown algae, a prevalent marine multicellular plant comprising species such as kelp, black-topped algae, sargassum, and Wakame, often grace people’s tables as a nutritional supplement due to their rich content of vitamins (chlorophyll A, carotene, and lutein) and inorganic salts [16,17]. Fucoidan (FCD), extracted from brown algae, is a highly branched, non-toxic water-soluble polysaccharide [18], consisting primarily of fucose, monosaccharide residues, sulfate groups, and small amounts of neutral monosaccharides, uronic acid, proteins, and phenolic compounds [19,20]. FCD has garnered significant interest for its natural antioxidant properties and its role in enhancing immunity. It has been extensively studied for its anticoagulant, antitumor, antiviral, and antioxidant effects [21,22,23,24]. Animal studies have shown that FCD, particularly in moderate and high doses, actively boosts the activity of the spleen and thymus [25]. Additionally, it modulates inflammatory factors via the NF-*κ*B pathway, helping to mitigate inflammation in the mouse liver [26]. The antioxidant efficacy of FCD is largely attributed to its lower molecular weight and higher sulfate content. Despite extensive studies on its structure and biological functions, research into FCD’s potential to alleviate histamine-induced liver and intestinal damage in mice remains limited.

This study aimed to assess the therapeutic effects of FCD on liver and intestinal damage induced by histamine in mice. This includes analyzing the liver enzyme levels, oxidative stress markers, examining liver tissues, monitoring blood inflammatory factors, exploring FCD’s therapeutic pathways at the protein level, and evaluating changes in the gut microbiota. The outcomes will provide a theoretical foundation for using fucoidan in health food and medicinal applications, underscoring its potential in developing prebiotics and hepatoprotective products. This research also holds practical value for enhancing the utilization of domestic brown algae resources and advancing their functional product development.

## 2. Materials and Methods

### 2.1. Chemicals and Reagents

Histamine (CAS#: 51-45-6) and silymarin (CAS#: 65666-07-1) were purchased from Beijing Pinellia Technology Development Co., Ltd. (Beijing, China) with purities of 99% and 80%, respectively. FCD (CAS#: 9072-19-9) with a purity of 95% and a molecular weight of 220–260 kD was obtained from Sigma (Shanghai, China). Paraformaldehyde (CAS#: 30525-89-4) and phosphate-buffered saline (PBS) were sourced from Shanghai Aladdin Bio-Chem Technology Co., Ltd. (Shanghai, China). Aspartate aminotransferase (AST), alanine aminotransferase (ALT), triglyceride (TG), MDA, GSH, and superoxide dismutase (SOD) assay kits were acquired from Nanjing Jiancheng Bioengineering Institute (Nanjing, China). ELISA kits for IL-1*β*, IL-6, and tumor necrosis factor (TNF-*α*) were purchased from Enzyme-linked Biotechnology (Shanghai, China), Dakewe Biotech (Beijing, China), and Nanjing Jiancheng Bioengineering Institute (Nanjing, China), respectively. LPS and 5-hydroxytryptamine (5-HT) were obtained from Jiangsu Meimian Industrial Co., Ltd. (Yancheng, China). The monoclonal antibody against *β*-actin was obtained from Wuhan Doctoral Biotechnology Co., Ltd. (Wuhan, China). The phospho-specific antibody against AKT was purchased from Cell Signaling Technologies (Beverly, MA, USA). Antibodies against TGF-*β*1, AKT, CYP2E1, Grp78, NLRP3, c-caspase1, and GSDMD were from Wuhan Sanying Biotechnology Co., Ltd. (Wuhan, China). All other reagents used were of analytical grade.

### 2.2. Animals and Experimental Treatment

Sixteen male C57BL/6 mice (6–8 weeks old) were procured from Weitong Lihua Experimental Animal Technology Co., Ltd. (Beijing, China). The mice underwent a week of adaptive feeding before the commencement of the experiment. Standard laboratory conditions were maintained, with a temperature of 22 ± 2 °C, relative humidity of 65% ± 5%, and a 12-h light–dark cycle [27]. The fodder was sourced from the Beijing Vital River Laboratory Animal Center [SCXK(Jing)2016-0006]. The material of fodder is safe and stable, providing balanced nutrition that meets the maintenance needs of experimental mice and minimizes raw material-related experimental errors. The ingredients include corn, wheat, and soybean meal, with a small quantity of fish meal, chicken meal, soybean oil, feed-grade sodium chloride, vitamin A, and vitamin D3. Following the adaptation period, the mice were randomly divided into four groups, each comprising four mice [28]: (1) CON group (control group), (2) MOD group (model group), (3) SIL group (silymarin treatment group), and (4) FCD group (FCD intervention group). Except for the CON group, the other three groups were administered histamine (15 mL/(kg·BW)) [6] for two weeks, while the CON group received an equivalent concentration of physiological saline. Subsequently, the SIL group and FCD group were treated with silymarin (63 mg/kg) [27] and FCD (100 mg/kg) [29], respectively, for an additional two weeks. Based on the experience of H. Li [29] and Abdel-Daim [30], the choice of FCD dosage was made with slight modifications. The CON and MOD groups continued receiving physiological saline of equal concentrations. The initial weight of the mice was recorded before the intervention (week 0), and subsequent weight measurements were conducted once a week for a total of five weeks. All of the animal studies were carried out according to the Ethics Review Committee of Laboratory Animal Welfare and Animal Experiments of China Agricultural University (permit number: Aw12402202-5-1; date of approval: 10 January 2022).

After four weeks of treatment, fresh feces were collected in sterile sampling tubes and promptly stored at −80 °C for subsequent analysis. Following a 12-h fasting period, all mice were anesthetized in a chamber filled with ether and euthanized. Tissue and blood samples were obtained: blood samples were collected in endotoxin-free tubes, immediately centrifuged (4 °C, 3000× *g* for 10 min) [14], and stored at −80 °C for further analysis. Liver tissues and ileum tissues were collected and rinsed in pre-cooled normal saline to remove the serum. The organs were then weighed and divided into three parts. One part was immersed in a preconfigured 4% (*v*/*v*) paraformaldehyde solution (prepared in PBS), another part was stored at −80 °C for subsequent analysis, and the remaining part was homogenized in nine times 0.9% pre-cooled normal saline to prepare a 10% (*w*/*v*) homogenate [22]. The homogenate was rapidly ground using a glass homogenizer in an ice-cold bath, followed by centrifugation at 12,000× *g* at 4 °C for 15 min. The resulting supernatant was collected and stored at −80 °C for later use.

**Figure 1:**
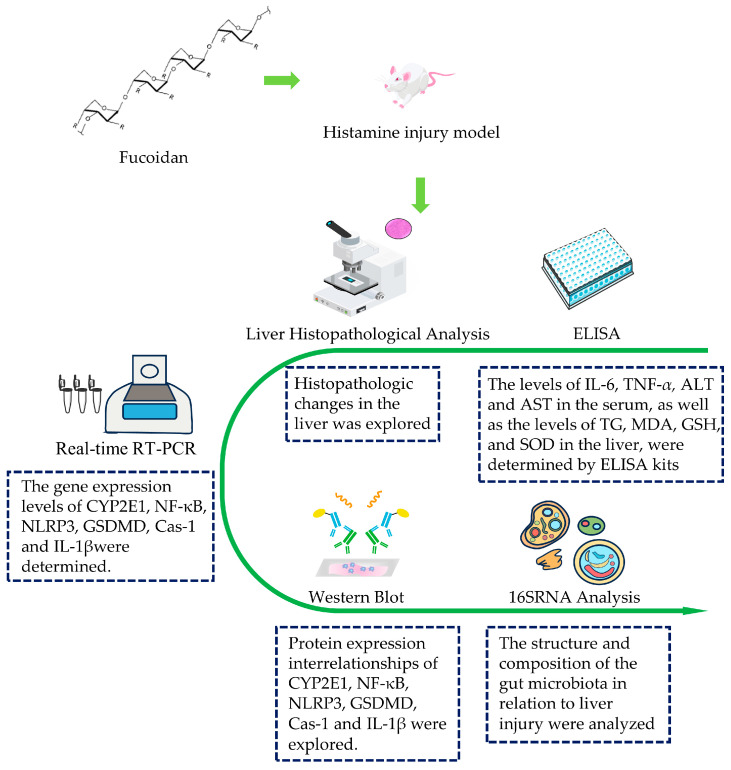
Flowchart of the methodology.

The flowchart of the methodology used in this study is summarized in Figure 1.

### 2.3. Determination of Serum Cytokines

The serum, obtained after centrifugation, was subjected to analysis to measure the levels of IL-6 and TNF-*α* using ELISA kits, following the manufacturer’s instructions.

### 2.4. Liver Function Test

The levels of ALT and AST in the serum as well as the levels of TG, MDA, GSH, and SOD in the supernatant of the liver homogenate were determined according to the instructions provided with the respective assay kits.

### 2.5. Liver Histopathological Analysis

Liver tissues fixed in 4% (*v*/*v*) paraformaldehyde solution were subjected to dehydration in graded alcohol and subsequent embedding in paraffin. Sections of approximately 5 μM thickness were stained with hematoxylin and eosin (H&E) for histopathological examination using a panoramic microscope (3DHITECH PANORAMIC 250, Debrecen, Hungary).

### 2.6. RNA Extraction and Quantitative Real-Time-Polymerase Chain Reaction (qRT-PCR)

#### Analysis

RNA was extracted from the liver tissue using the High Purity Total RNA Rapid Extraction Kit (Tiangen Biotech Co., Ltd., Beijing, China). Reverse transcription was performed with the ReverTra Ace^®^ qPCR RT Master Mix Kit (Tiangen Biotech Co., Ltd., Beijing, China). cDNA was obtained from the liver tissue, and PCR amplification was performed according to the instructions of the TOYOBO SYBR^®^ Green Realtime PCR Master Mix Kit under preset conditions. SuperReal PreMix Plus (Tiangen Biotech, Beijing, China) was used for real-time fluorescent quantitative PCR analysis. All analyses were conducted in triplicate, with normalization of the relative expression levels of target gene mRNAs by the glyceraldehyde-3-phosphate dehydrogenase (GAPDH) mRNA levels. The results were presented using the 2^−ΔΔCt^ method, and primer sequences are listed in Table 1.

### 2.7. Western Blotting

The liver samples were homogenized and lysed using RIPA buffer supplemented with polymethylsulfonyl fluoride (PMSF). After assessing the total protein concentration using the BCA protein assay kit, 30 μg of protein from each sample was solubilized through 12% SDS-PAGE. The protein strips were electrotransferred onto a PVDF membrane from Solarbio Science & Technology in Beijing, China. After incubating with 5% skim milk powder for 1 h to block non-specific sites, the membrane was further incubated with the primary antibody at 4 °C overnight. After incubation with the primary antibody, the membrane underwent two washes with TBS and was subsequently incubated with a secondary antibody coupled with horseradish peroxidase at a dilution of 1:1000 or 1:800 for one hour. Detection was performed through the utilization of an ECL Western Blotting Kit (BioVision, San Francisco, CA, USA). The relative density of the protein bands was evaluated using the Bio-Rad Gel Doc 2000 image analysis system (Bio-Rad, Hercules, CA, USA).

### 2.8. Determination of Gut Tissues

The levels of LPS, IL-1*β*, and 5-HT in the gut tissues were quantified using ELISA assay kits. The experimental procedures were performed according to the instructions provided by the supplier.

### 2.9. Analysis of the Gut Microbiota

Before euthanizing the mice, fresh feces were collected and stored in sterile tubes at −80 °C for future analysis of the structure and composition of the gut microbiota via 16S rRNA gene sequencing. Total DNA was extracted from the fecal samples. The universal primer pairs 338F and 806R were used to amplify the V3–V4 region of the 16S rDNA gene by PCR [31]. PCR product sequencing was conducted using the Illumina MiSeq platform at Majorbio Bio-Pharm Technology Co., Ltd., located in Shanghai, China. We utilized UPARSE (version 7.0.1090) for the bioinformatics analysis of operational taxonomic units (OTUs) at a similarity level of 97% and RDP Classifier version 2.2 for annotating the OTU sequences with a classification, using a confidence threshold of 0.7 [32]. Visual representation of the compositional similarity and overlap of environmental samples at different classification levels, particularly at the OTU level, can be achieved using Venn diagrams. Through the analysis of diversity indices, we can gather information on species richness, diversity, and coverage within a community. Our research primarily focused on the ACE index, Chao richness, and Shannon and Simpson diversity indices. At the OTU level, beta diversity was analyzed using principal component analysis (PCA) and non-metric multidimensional scale analysis (NMDS). The study utilized the linear discriminant analysis effect size (LEfSe) method to identify variations in microbial community abundance across different groups, with an LDA score of 4.0 [33]. The correlation between microbial classification and intestinal LPS, IL-1*β*, and 5-HT was assessed using Spearman correlation analysis [34].

### 2.10. Statistical Analyses

The results were expressed as the mean ± standard deviation (SD) and analyzed using GraphPad Prism 8.0 software (GraphPad Software, San Diego, CA, USA). The data were evaluated using one-way analysis of variance (ANOVA) and the LSD method. Statistical significance was considered when *p* < 0.05.

## 3. Results

### 3.1. Effects of FCD on Organ Index and Serum Cytokine Secretion in Mice

**Figure 2:**
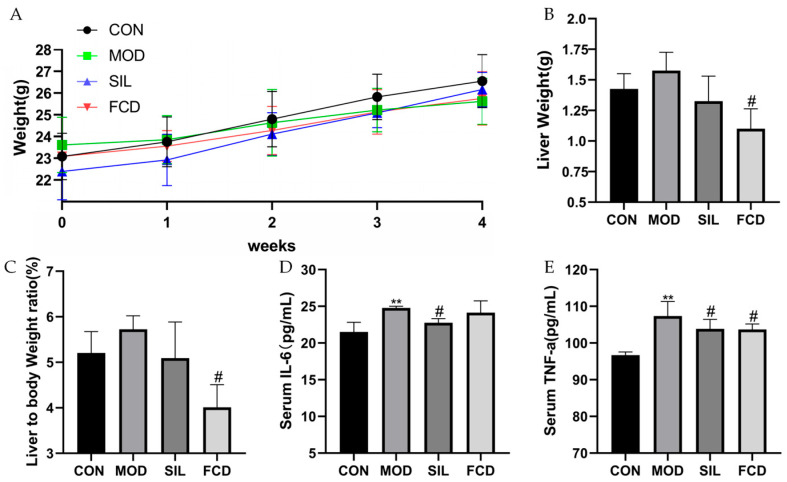
The effect of FCD on organ index and inflammatory cytokines in the serum of mice. ** *p* < 0.01 vs. CON; ^#^ *p* < 0.05 vs. MOD. (*n* = 4). (**A**) Changes in body weight, (**B**) liver weight, (**C**) liver organ index, (**D**) serum IL-6, and (**E**) serum TNF-*α*.

To evaluate the impact of FCD, we monitored the weekly body weights, observing no significant initial differences among the groups (23 ± 1 g). After four weeks, all groups showed a weight increase without statistical variation (Figure 2A, *p* > 0.05). The liver organ index notably increased in the model group, suggesting mild liver swelling induced by histamine, which was somewhat mitigated by FCD (Figure 2B,C, *p* < 0.01).

Histamine significantly raised the serum IL-6 and TNF-*α* levels in the model group compared to the control group (Figure 2D,E, *p* < 0.01). FCD treatment effectively lowered the TNF-*α* levels (*p* < 0.05), while the IL-6 levels remained unchanged (*p* > 0.05), indicating FCD’s potential to modulate inflammatory responses.

### 3.2. Effect of FCD on Liver Function

**Figure 3:**
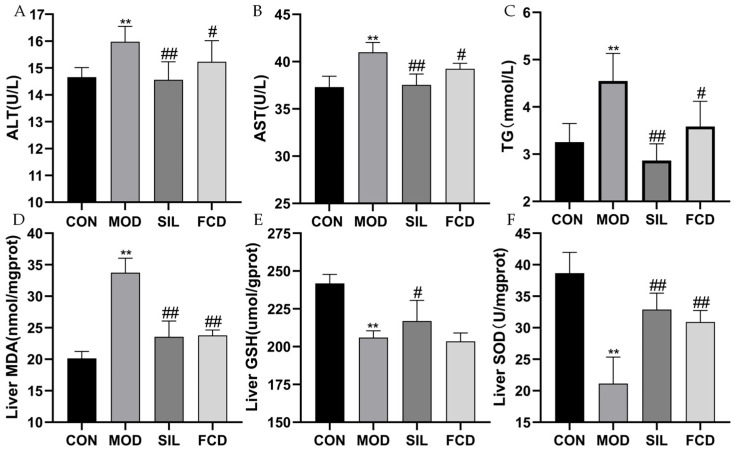
The effect of FCD on liver function indices and levels of oxidative stress of mice. ** *p* < 0.01 vs. CON; ^#^ *p* < 0.05, ^##^ *p* < 0.01 vs. MOD. (*n* = 4). (**A**) ALT, (**B**) AST, (**C**) TG, (**D**) liver MDA, (**E**) liver GSH, and (**F**) liver SOD.

Assessment of the ALT, AST, and TG levels showed significant increases in the model group compared to the control, indicating liver damage (1.09-fold, 1.1-fold, and 1.29-fold, respectively; Figure 3A–C, *p* < 0.01). Both the silymarin and FCD treatments reduced these markers (*p* < 0.05), suggesting their hepatoprotective effects. The model group also showed increased oxidative stress, evident from the elevated MDA levels and reduced GSH and SOD levels (Figure 3D–F, *p* < 0.01). Both treatments improved these parameters, particularly FCD, which significantly reduced the MDA levels and increased the SOD activity (*p* < 0.01), underscoring its antioxidative potential.

### 3.3. Histological Analysis of Liver

**Figure 4:**
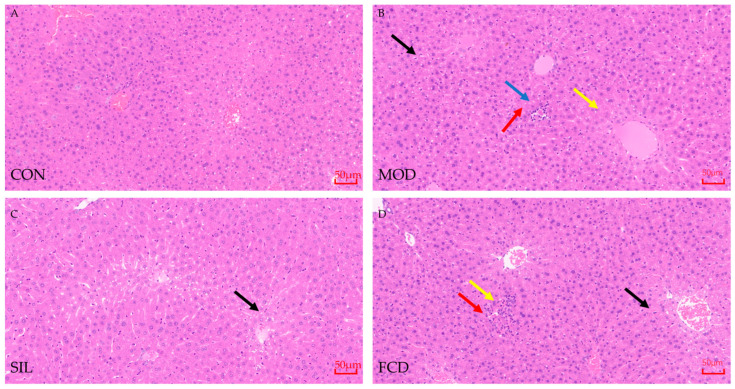
The histological sections of mice liver. (**A**–**D**) represents CON group, MOD group, SIL group, and FCD group, respectively. The black arrow represents the circular voids, the blue arrow represents hepatocyte necrosis and karyolysis, the yellow arrow represents watery degeneration, cellular swelling, and loosely stained cytoplasm in the liver slices, and the red arrow represents the infiltration of lymphocytes.

Liver slices from mice underwent histological examination (Figure 4). In the CON group, liver cells were well-organized with intact structures, showing no signs of inflammation or necrosis. No apparent dilation of venous blood vessels or hepatic sinus congestion was observed. In contrast, the MOD group exhibited hepatocytes with watery degeneration, cellular swelling, and loosely stained cytoplasm (yellow arrow). Localized hepatocyte necrosis and karyolysis (blue arrow), infiltration of lymphocytes (red arrow), and mild steatosis with small circular vacuoles in the cytoplasm (black arrow) were evident. Conversely, the SIL group demonstrated well-organized cells with minimal signs of inflammation, whereas in the FCD group, there was only slight lymphocyte infiltration and significant restoration of cell swelling. These findings suggest that FCD has significant potential to reduce the inflammation, cell infiltration, and necrotic damage that histamine induces.

### 3.4. Effect of FCD on mRNA Expression Levels of Liver-Related Proteins

**Figure 5:**
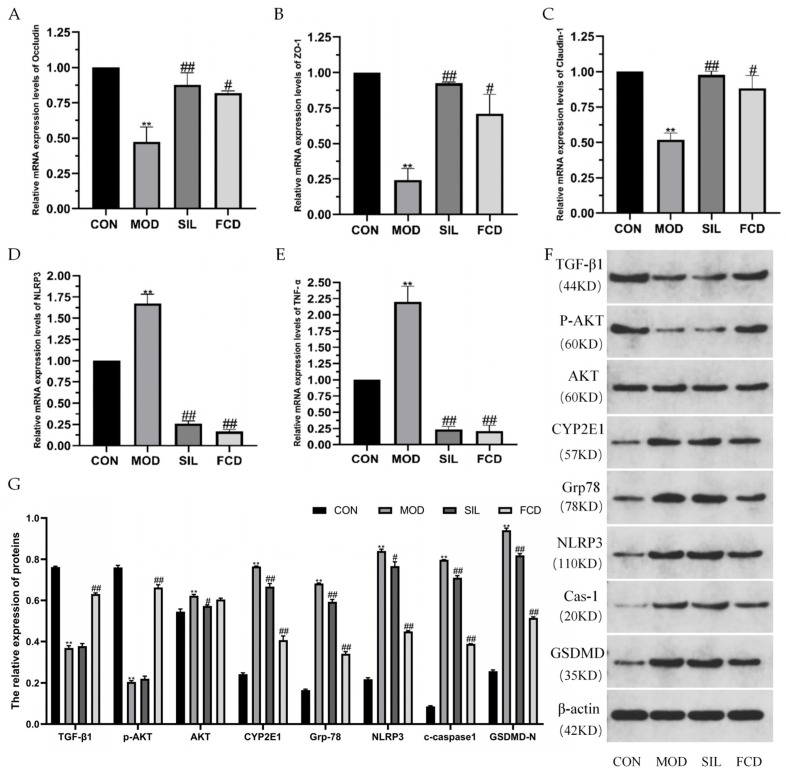
The effect of FCD on mRNA expression levels and protein expression levels in the liver. ** *p* < 0.01 vs. CON; ^#^ *p* < 0.05, ^##^ *p* < 0.01 vs. MOD. (*n* = 4). (**A**) Occludin, (**B**) ZO-1, (**C**) Claudin-1, (**D**) NLRP3, (**E**) TNF-*α*, (**F**) Western blot analysis of the expression of TGF-*β*1, *p*-AKT, AKT, CYP2E1, Grp78, NLRP3, Cas-1, and GSDMD in liver tissues, and (**G**) the relative expression of proteins.

As shown in Figure 5A–E, the mRNA expression levels of tight junction proteins (Occludin, ZO-1, and Claudin-1) in mouse livers were significantly reduced (*p* < 0.01) following histamine treatment compared to the CON group, indicating a disruption of the hepatic barrier. Concurrently, there was an increase in the expression levels of NLRP3 and TNF-*α* (*p* < 0.01), suggesting the initiation of liver lesions and inflammation induced by histamine. SIL and FCD treatment significantly increased the mRNA expression levels of Occludin, ZO-1, and Claudin-1 (*p* < 0.05) and decreased the NLRP3 and TNF-*α* levels (*p* < 0.01), indicating the therapeutic potential of FCD similar to SIL. These results highlight the therapeutic potential of FCD, similar to that of SIL, in effectively mitigating liver damage and slowing the progression of histamine-induced liver fibrosis and functional impairment.

### 3.5. Effects of FCD on the Protein Expression Levels in the Liver

The results of the Western blot analysis (Figure 5F,G) indicate that histamine feeding suppressed the expression of TGF-*β*1 and *p*-AKT. However, treatment with FCD significantly reversed the effects of histamine on the liver TGF-*β*1 and *p*-AKT levels. Additionally, the levels of AKT, CYP2E1, and Grp78 were higher in mice in the MOD group than in the CON group (*p* < 0.05 or *p* < 0.01). The levels of AKT, CYP2E1, and Grp78 were significantly downregulated in mice in the FCD group compared to those in the MOD group (*p* < 0.05). It is suggested that treatment with FCD could inhibit histamine-induced liver injury. Furthermore, FCD also alleviated the abnormal elevation of NLRP3, Cas-1, and GSDMD induced by histamine, thus repairing the liver by inhibiting the NLRP3-Cas-1-GSDMD inflammatory vesicle signaling pathway. This suggests a protective role of FCD in reducing inflammation and promoting liver recovery in histamine-challenged mice.

### 3.6. Effect of FCD on Intestinal Cytokine Secretion

**Figure 6:**
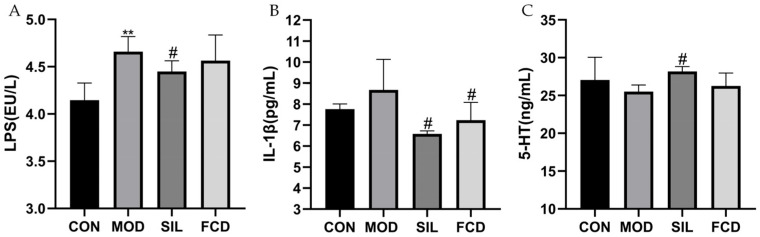
The effect of FCD on gut LPS, IL-1*β*, and 5-HT. ** *p* < 0.01 vs. CON; ^#^ *p* < 0.05 vs. MOD. (*n* = 4). (**A**) LPS, (**B**) IL-1*β*, and (**C**) 5-HT.

In Figure 6, the MOD group exhibited a significant increase in intestinal LPS levels compared to the CON group (*p* < 0.01), indicating possible damage to the intestinal mucosal barrier and a disruption of intestinal balance. The SIL group exhibited a pronounced reduction in intestinal LPS (*p* < 0.05). In contrast, FCD treatment did not demonstrate a significant inhibitory effect on the intestinal LPS levels. Additionally, the levels of mouse intestinal IL-1*β* and 5-HT in each group did not fluctuate significantly. These results suggest that FCD may not exert a significant impact on intestinal LPS levels, but does not cause a significant change in the IL-1*β* and 5-HT levels in the intestine.

### 3.7. Influence of FCD on the Gut Microbiota of Mice

**Figure 7:**
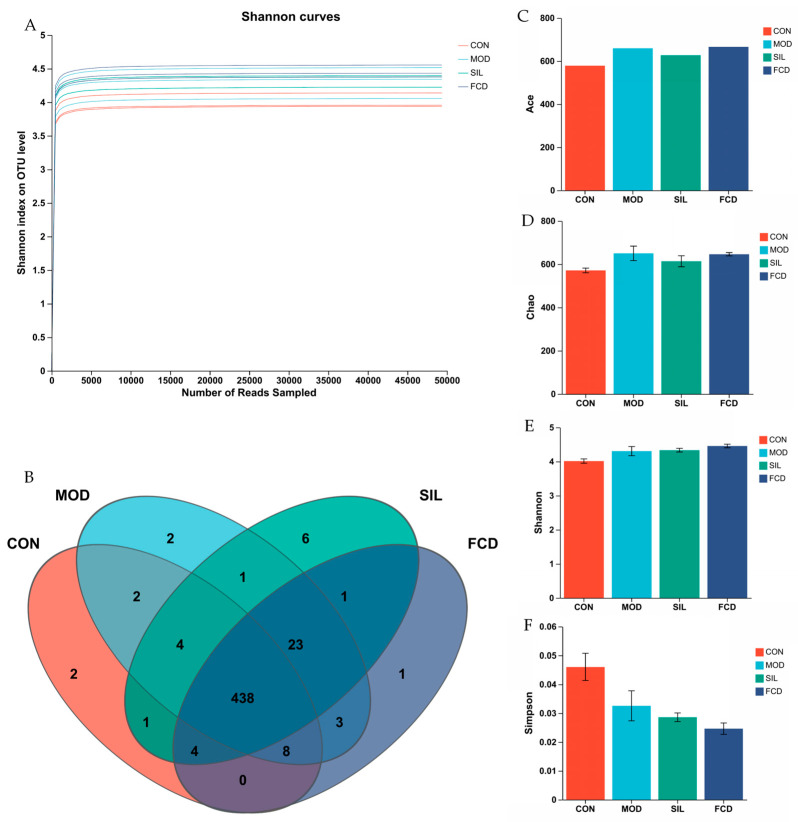
The effect of FCD on gut microbiota (a). (**A**) Rarefaction curves of OTUs clustered at 97% sequence similarity across different groups, (**B**) OTU Venn diagram, (**C**) Ace index, (**D**) Chao index, (**E**) Shannon index, (**F**) Simpson index.

To investigate the impact of FCD on the gut microbiota composition in mice, 16S rRNA gene sequencing was conducted on the fecal samples. After excluding unqualified and invalid sequences, a total of 888,705 valid reads were obtained, ensuring sufficient sample coverage and species richness (Figure 7A). Operational taxonomic units (OTUs) were employed to evaluate the species composition within each group by clustering high-quality sequences at 97% similarity. The number of OTUs identified in the CON, MOD, SIL, and FCD groups were 459, 481, 468, and 478, respectively (Figure 7B). Alpha diversity metrics including Ace and Chao1 for richness and the Shannon and Simpson indices for diversity showed no significant differences between the groups (Figure 7C–F, *p* > 0.05), suggesting that neither FCD feeding nor histamine treatment markedly affected the richness or diversity of the gut microbiota.

**Figure 8:**
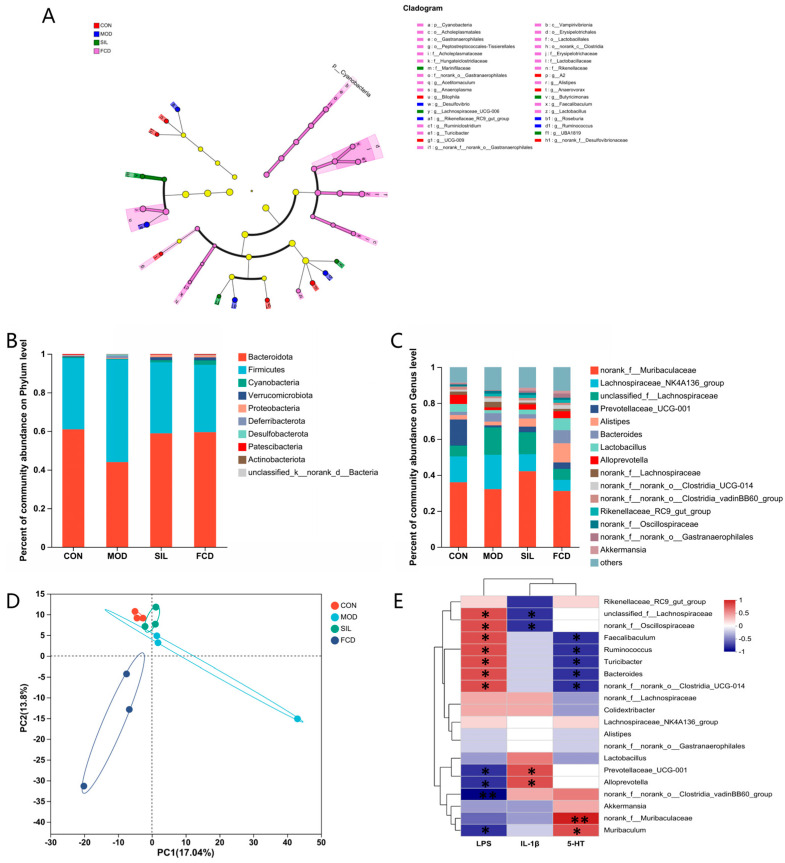
The effect of FCD on gut microbiota (b). (**A**) Biological cladograms. (**B**) The relative abundance plot in the bacterial phylum level of the gut microbiota. (**C**) The relative abundance plot in bacterial genera of the gut microbiota. (**D**) PLS-DA analysis diagram of the beta diversity intestinal flora structure of different samples. (**E**) Spearman correlation analysis of the gut microbiota, LPS, IL-1*β*, and 5-HT. * *p* < 0.05, ** *p* < 0.01 (*n* = 4).

Further species-level analysis using LEfSe confirmed these findings (Figure 8A). Structural and compositional changes in the microbial communities were evaluated at the phylum and genus levels based on the sequence data. *Bacteroides* and *Firmicutes*, dominant phyla in the mammalian gastrointestinal tract, accounted for a substantial proportion. Among the 22 microbial communities with relative abundances greater than 1% identified at the genus level, dominant genera included *norank_F_Muribaaculaceae*, *Lachnospiraceae_NK4A136_Group*, *unclassified_F_Lachnospiraceae*, *Prevotelaceae_UCG-001*, and *Alistipes*, among others. Compared to the MOD group, the FCD group exhibited a reduced relative abundance of *norank_F_Muribaaculaceae*, *Lachnospiraceae_NK4A136_Group*, and *unclassified_F_Lachnospiraceae* while showing an increased relative abundance of *Prevotelaceae_UCG-001* and *Alistipes*. Additionally, the FCD group showed an increased abundance of *Lactobacillus* compared to the other groups (Figure 8B,C).

Diversity analysis (Figure 8D) and principal component analysis (PCA) indicated that the gut microbiota of the CON and SIL groups exhibited similarity, while the MOD group displayed distinct compositional changes compared to the CON group, likely attributed to histamine-induced alterations. Mice fed with FCD demonstrated significant changes in their gut microbiota, characterized by a unique microbial composition, suggesting the potential of FCD to modulate the gut microbiota and enhance its therapeutic effects.

Spearman correlation analysis provided a heatmap illustrating the relationships between the gut microbiota and intestinal biomarkers LPS, IL-1*β*, and 5-HT (Figure 8E). At the genetic level, the relative abundance of the *Rikenellaceae_RC9_gut_group* was negatively correlated with the level of IL-1*β* (*p* < 0.05). The relative abundance of *unclassified_f__Lachnospiraceae* and *norank_f__Oscillospiraceae* was positively correlated with the level of LPS and negatively correlated with the level of IL-1*β* (*p* < 0.05). *Faecalibaculum*, *Ruminococcus*, *Turicibacter*, *Bacteroides*, and *norank_f__norank_o__Clostridia_UCG-014* were positively correlated with LPS and negatively correlated with 5-HT (*p* < 0.05). *Prevotellaceae_UCG-001* and *Alloprevotella* showed a negative correlation with LPS and a positive correlation with IL-1*β* (*p* < 0.05). *Muribaculum* was negatively correlated with the level of LPS and positively correlated with 5-HT (*p* < 0.05). Moreover, an increase in the relative abundance of *norank_f__norank_o__Clostridia_vadinBB60_group* significantly decreased the LPS levels (*p* < 0.01), while an increase in *norank_f__Muribaculaceae* elevated the 5-HT levels (*p* < 0.01). These findings underscore the complex interactions between FCD treatment and gut microbiota dynamics, indicating its influence on gut health and systemic inflammatory responses.

## 4. Discussion

This study investigated the potential alleviating effects of FCD on histamine-induced discomfort, focusing on its impact on liver, intestinal health, and systemic balance in mice. Mice were administered histamine, a potentially harmful component often present in fermented foods, to induce discomfort, followed by treatment with FCD to evaluate its therapeutic effects. After 28 days, results indicated that FCD effectively mitigated liver injury, regulated intestinal microbiota, and influenced metabolic pathways.

In the context of assessing the therapeutic effects of FCD on body weight and liver function in histamine-treated mice, our findings revealed that neither histamine nor FCD exerted a significant impact on the weight of the experimental mice. However, an observed slight increase in the liver organ index of mice subjected to the histamine group suggests potential liver damage, manifesting as edema and congestion. Notably, supplementation with FCD demonstrated the capacity to ameliorate this effect. Histamine exposure is known to induce the upregulation of endotoxin, triggering the activation of Kupffer cells and subsequently leading to the excessive production of inflammatory factors [35]. These inflammatory factors play a crucial role in maintaining tissue homeostasis, orchestrating processes such as inflammation, cell death, proliferation, migration, and healing mechanisms. Among the key mediators of inflammation, IL-6 has been identified as a major player [36], while TNF-*α* stands out as a significant pro-inflammatory cytokine capable of inducing the secretion of other cytokines and enzymes by various cells and tissues [1]. To further elucidate the potential inhibitory effects of FCD on these inflammatory factors, we conducted an analysis of the IL-6 and TNF-*α* levels in the blood of the mice. While FCD did not exhibit a significant intervention effect on the IL-6 levels, it demonstrated a noteworthy reduction in the abnormally elevated levels of TNF-*α* in the blood. This suggests that FCD exerts a certain inhibitory effect on the secretion of inflammatory factors in the serum, thereby indicating a potential enhancement of immune activity.

Furthermore, FCD exhibited a pronounced restorative effect on impaired liver function, as evidenced by a significant reduction in the serum levels of transaminases including AST and ALT. Typically present at low levels in the serum, AST and ALT become elevated when liver damage occurs, making them valuable indicators of liver health [37,38]. Our experiments demonstrated that FCD plays a role in alleviating liver injury by reducing transaminase levels in the bloodstream. MDA, a by-product of lipid peroxidation, was also investigated in our study. Elevated MDA levels can interfere with antioxidant effects, contributing to liver cell degeneration and necrosis [29]. In contrast, GSH and SOD are essential components of the body’s antioxidant system, playing a crucial role in protecting the liver against oxidative stress damage [39]. SOD, an antioxidant metalloenzyme, catalyzes the breakdown of superoxide anion radicals, converting reactive oxygen species into hydrogen peroxide, which is further decomposed into water by GSH. Our experiments demonstrated that FCD effectively regulated various oxidative stress markers in the liver, contributing to the maintenance of liver health. Pathological assessment of the liver slices further supported the therapeutic potential of FCD. The observed improvement in liver lesions, characterized by reduced inflammation and the alleviation of liver cell infiltration and necrosis, underscored the ability of FCD to mitigate histamine-induced damage [30]. These comprehensive findings emphasize the multifaceted protective effects of FCD on liver function, positioning it as a potential therapeutic agent for addressing liver injury and oxidative stress.

The qPCR results provide valuable insights into the molecular mechanisms underlying the therapeutic effects of FCD on histamine-induced liver damage. The upregulation of critical protein genes including Occludin, ZO-1, and Claudin-1 indicates a potential correlation between FCD treatment and the amelioration of liver lesions. By administering a specific dosage of FCD, the expression of inflammation-related genes such as NLRP3 and TNF-*α* was reduced to their usual levels, suggesting that FCD plays a role in impeding the conversion of stellate cells to fibroblasts and reducing liver fibrosis. The qPCR results further demonstrated that FCD treatment ameliorated liver barrier destruction caused by histamine, leading to a partial restoration of normal liver function.

At the protein level, our study delved into the hepatic repair capacity of FCD. TGF*β*1, a group of protein polypeptides with multiple biological functions, was found to be regulated by FCD, contributing to the reduction in hepatic fibrosis [40]. Additionally, FCD exhibited inhibitory effects on AKT activation in the liver, thereby blocking downstream signaling regulated by AKT. Furthermore, our investigation into the expression of CYP2E1 revealed that FCD significantly inhibited histamine-induced CYP2E1 expression at the protein level. This is particularly relevant given CYP2E1’s involvement in the metabolism of various endogenous and exogenous substances in organisms, primarily in the liver, intestine, and heart. Histamine-induced overexpression of TXNIP, which converts TXNIP from thioredoxin to an activator of the NLRP3 inflammasome, was addressed by FCD. This led to the normalization of the NLRP3 levels, thus providing a consistent conclusion across both gene and protein expression analyses. Moreover, FCD demonstrated an inhibitory effect on GSDMD, a component present in nigericin-induced NLRP3 inflammasomes. These inflammasomes play a role in inflammatory activation sites, participating in the inflammatory response. NLRP3 exacerbates inflammation in the liver by activating Cas-1 and cleaving precursor forms of IL-18 and IL-1*β*. FCD achieves its inhibitory effect on TXNIP overexpression by targeting the NLRP3-Cas-1-GSDMD inflammatory vesicle signaling pathway.

Liver health is intricately linked to intestinal well-being, where the intestinal environment, comprising bacteria, bacterial endotoxins, and digestion and absorption products, significantly influences liver metabolism. Histamine, known for its damaging effects on the liver, also disrupts intestinal integrity. Consequently, our experiment delved into exploring FCD regulatory impact on the intestine, considering its critical role in food digestion, nutrient absorption, and the preservation of intestinal stability. The intestinal mucosa, a vital barrier shielding the intestine, plays a crucial role in preventing disruption by harmful microorganisms or endotoxins and maintaining intestinal homeostasis [41]. Histamine-induced damage to the intestinal mucosa not only disrupts intestinal balance but may lead to systemic inflammation. Among the various endotoxins, LPS produced by Gram-negative bacteria activates pro-inflammatory cytokine cascades, contributing to the production of pro-inflammatory cytokines through plasma membrane proteins like TLR4 and CD14.

Our experiment noted a significant increase in LPS levels in mice fed with histamine, which likely contributed to inflammation. While FCD did not significantly reduce the intestinal LPS levels in this study, it suggests potential avenues for future research, particularly at higher dosages, to explore its full effects. Notably, after histamine ingestion, there were disturbances in the IL-1*β* levels in the intestine, which FCD effectively mitigated, demonstrating its capacity to modulate inflammatory responses. Furthermore, changes in the 5-HT levels, which affect sleep and appetite, did not show significant differences among the groups, indicating that under the conditions tested, FCD may not substantially influence the 5-HT levels. This outcome highlights the need for further studies to delineate FCD’s impact on neurotransmitter levels more clearly. This study illuminates the complex interactions between histamine-induced disruptions, intestinal integrity, and the potential regulatory effects of FCD. Although the modulation of LPS levels by FCD did not achieve statistical significance, the observed effects on IL-1*β* provide valuable insights into its anti-inflammatory potential. These findings enhance our understanding of FCD’s role in mitigating histamine-related disturbances and maintaining intestinal balance, pointing to its promising therapeutic applications. Further investigations into the dosage and impact on additional biomarkers are warranted to fully characterize FCD’s therapeutic potential and optimize its application in health food and medicinal products.

FCD, being a natural polymer compound, exhibits notable resistance to degradation within the human digestive system. The majority of FCD remains resilient to breakdown, allowing only a small fraction to enter the bloodstream. The predominant influence of FCD is directed toward the host’s intestinal cavity, where it interacts with intestinal microorganisms and their metabolites [42]. The intimate relationship between the gut microbiota and the intestine forms a crucial connection that plays a pivotal role in maintaining overall health. In our experimental approach, the relative abundance of gut microbiota was quantified using OTUs. At the phylum level, the dominant phyla observed were *Bacteroides*, *Firmicutes*, *Verrucomicrobiota*, *Cyanobacteria*, and *Proteobacteria*. A comparative analysis with the MOD group revealed that FCD induced a reduction in Firmicutes and an increase in *Bacteroides*, indicating the therapeutic and palliative effects of FCD in regulating the dominant bacterial composition in the intestines of histamine-treated mice. Delving deeper at the genus level, FCD showcased a regulatory influence by reducing the relative abundance of *norank_F_Muribaaculaceae*, *Lachnospiraceae_NK4A136_Group*, and *unclassified_F_Lachnospiraceae*. Concurrently, there was an increase in the relative abundance of *Prevotelaceae_UCG-001* and *Alistipes*. This dynamic modulation promoted the stability of the gut microbiota, emphasizing FCD’s potential in fostering a balanced microbial community in the face of histamine-induced disruptions. The observed alterations in the relative abundance of specific bacterial taxa at both the phylum and genus levels underscore the regulatory impact of FCD on the gut microbiota. These findings contribute to a comprehensive understanding of how FCD may play a role in shaping and maintaining a resilient intestinal microbial ecosystem, particularly in the context of histamine-related challenges.

## 5. Conclusions

This study explored the therapeutic potential of fucoidan (FCD) in addressing disturbances induced by histamine in a murine model. Our investigation focused on the effects of FCD on liver injury, inflammation regulation, and gut microbiota modulation following histamine exposure. The results demonstrated that FCD effectively mitigated liver dysfunction and inflammation, evidenced by reduced levels of transaminases and lipid peroxidation, alongside an enhancement in antioxidant defenses.

Molecular analyses highlighted FCD’s significant impact on key proteins involved in liver barrier function and inflammatory response, suggesting a comprehensive protective mechanism against histamine-induced damage. Additionally, FCD exerted a regulatory influence on the gut microbiota, altering its composition to favor a healthier microbial balance, which further supports its role in maintaining overall intestinal and systemic health.

These findings underscore FCD’s multifaceted therapeutic potential in alleviating disturbances related to histamine exposure. They suggest that FCD not only protects against liver damage, but also modulates systemic inflammation and supports gut health, making it a promising candidate for further research and potential clinical applications.

Further studies involving higher dosages, varied administration frequencies, and additional physiological parameters are warranted to deepen the understanding of FCD’s mechanisms and explore its broader applications in health food and medicinal products. This research paves the way for the future exploration of FCD as a functional supplement or therapeutic agent in conditions associated with dietary imbalances and oxidative stress.

## Figures and Tables

**Table 1:** The primer sequences in RT-qPCR.

*Gene*	Forward Primer (5′-3′)	Reverse Primer (5′-3′)
*GADPH*	GGCAAGTTCAACGGCACAG	CGCCAGTAGACTCCACGACAT
*TNF-α*	GGAAAGGACGGACTGGTGTA	TGCCACTGGTCTGTAATCCA
*NLPR3*	GTGGTGACCCTCTGTGAGGT	TCTTCCTGGAGCGCTTCTAA
*Occludin*	GAGGAGAGTGAAGAGTACATGGGCTG	GTCTGTCATAATCTCCCACCATCCT
*Zonula occludens-1 (ZO-1)*	TCATCCCAAATAAGAACAGAGC	GAAGAACAACCCTTTCATAAGC
*Claudin-1*	TCCTTGCTGAATCTGAACA	AGCCATCCACATCTTCTG

## Data Availability

The original contributions presented in the study are included in the article, further inquiries can be directed to the corresponding author.
